# The Dentist

**Published:** 1896-08

**Authors:** B. F. Shepherd

**Affiliations:** Pleasantville, Ind.


### The Dentist.

BY DR. B. F. SHEPHERD, PLEASANTVILLE, IND.

Read before the Indiana State Dental Society, Indianapolis, Ind., June 30, 1896.

You will, no doubt, think it presumption in me to present to you a paper on this subject. (Granted, without argument.) But you will all agree with me, that with all his knowing, the dentist should know something of himself. While he is investigating everything within reach, he should take a look within, for there lies his storehouse of wealth—his fortune of strength and power.

Will you go with me, then, a little way, while I try to describe some of the physical, mental and moral traits which should characterize the dentist?

In the first place, he should be endowed with a harmonious temperament and good health. He should have an ample vital-system, so that he can replenish rapidly and abundantly the wastes of his system. This temperament gives cheerfulness, order and cordial magnetism, which carries sunshine everywhere. It also gives plumpness and a slight tendency to fleshiness. It gives a deep chest, with copious breathing-power; a rapid and abundant circulation, and makes a man hearty, zealous and enthusiastic.

He should have an ample amount of the motive temperament. This, when prominent, is indicated by a strong frame, prominent features, hair strong, and a rather dark complexion. He should also have the mental temperament in a pretty strong degree. This temperament gives an active mind, a studious disposition, a love of knowledge, and an interesting, fact-gathering, philosophical and inventive cast of mind. It is indicated by a clear, sharp eye, well-defined but somewhat delicate features, fine hair, fine skin, with comparatively small bones and muscles, large brain and general sprightliness of body, and an abundance of sensitiveness and susceptibility. When these temperaments are possessed in harmonious blending, when each is about equally represented in the man, there will be a good frame, with strength not amounting to coarseness; there will be refinement without effeminacy, and general strength, earnestness, health, endurance, and the basis of long life; in short, a well-organized man.

The life of the dentist is one of care, fatigue, patience, perseverance and self-denial. Hence he needs a temperament that lies at the basis of all these qualities. If he has an excess of the nervous or mental temperament he will become easily worn and anxious, irritable and unhappy; and he will meet his patients with a spirit of repulsion, making the weak and nervous more so, and the irritable still more disagreeable.

If he has too much of the motive temperament, there will be a lack of gentleness and refinement and taste. He will inspire fear rather than confidence, especially with the weak and delicate.

If he has too much of the vital temperament, there will be a lack of studiousness, a tendency to over-eat and live too highly, and thereby produce a muddy mind, an obtuse intellect and judgment, and a sort of grossness which will not be agreeable to persons of refinement and culture.

Nothing is more unfortunate in the practice of dentistry than qualities, constitutional or acquired, in the dentist, which make him ill-adapted to meet humanity, in its more sensitive and delicate phases, pleasantly. The dentist should not, certainly, in his own person, be offensive, yet he should have a world of strength, be hearty and cheerful, with sunshine enough for his own requirements and enough to brighten all with whom he comes in contact, especially in a professional way. The dentist should be so organized, mentally and physically, as not to be repulsive to the refined, yet he should have strength left to minister strength to the depressed and weak. In short, no man needs a better constiution or a more harmonious development than the dentist.

We now come to the inquiry as to his mental peculiarities. And we might say, in general, that perfect harmony and a strong development of all the mental qualties would be highly advisable, but as most men are not thus favorably organized, we will specify some of the indispensable elements.

In the first place, the dentist needs a world of knowledge of a practical character. He should understand anatomy, physiology (especially of the head and face); he should have a knowledge of chemistry, botany, mineralogy, pathology, surgery and mechanical dentistry. These sciences require a full development of the perceptives. The dentist must see or he can never know; he must be a judge of size, form, color, etc., or he will not be able to appreciate the conditions necessary in adjusting artificial dentures, in the alignment or regulation of abnormal conditions, and will fail in the general touch and finish of all his work.

He should also have a large memory, that he may hold all the knowledge he may get from books, from observation, and from experience. His reasoning faculties should be amply developed, so that he can analyze, discriminate, and comprehend the philosophy of the causes involved in a given case: the patient's peculiar temperament and conditions, and circumstances unlike anything he has seen before. Hence he must understand the philosophy involved in the facts. If he has only the perceptives well developed, he will just be able to do what he has seen done, while the case in hand may require very different treatment from anything he has seen. Hence he must fail, and will not be able to strike out any new lines for himself. What he needs is the faculties of the fact-gatherer and the philosopher, with constructiveness sufficient to put facts or materials together creditably.

The dentist should be a man of decision and self-reliance; he should feel that he knows, and is able to do, so that his very attitude will forbid the suggestions of those who will have everything their own way and still hold him responsible for the success of the operation. Many a man who really knows fails because of a lack of self-reliance, thoroughness and stamina equal to his knowledge. He should cultivate that spirit of fortitude which will enable him to grapple with any and all difficulties bravely, decisively, marking the hand of a master in all his work.

The dentist should have combativeness and destructiveness sufficient to give efficiency; enabling him to witness pain and suffering and to employ the means necessary to relieve without trembling or hesitancy. It has been said that the physician needs the lion's heart and the woman's hand. It is equally true of the dentist—he should have combativeness and destructiveness, firmness and self-esteem, to give the lion-like power—ideality, constructiveness and gentleness, which come from refinement of temperament, to give the woman's hand. We have seen men who had power, self-reliance and persistency, who carried these forces with admirable gentleness and self-control.

He should have prudence, caution, secretiveness and good common sense, and the more common sense the better. Cautiousness will make him desire to do the right thing at the right time and place. Secretiveness will enable him to keep his mouth shut at the proper time, and control his countenance as well as his expressions, and be always master of the situation, when too open an expression would have been disastrous.

He should have large hope and mirthfulness, and an excellent talking talent, so that he may put an atmosphere of mirthfulness and good feeling around him at all times.

Large constructiveness is necessary to the dentist, in fact his profession has been considered by some as only a mechanical trade. While this is not true, yet his ability to put together, his judgment or knowledge of the right conditions in constructions, will mark his success in prosthetic and operative dentistry, as well as all operations in oral surgery. It will enable him to meet the requirements of anæsthetics in dentistry, elaborating and bringing together the many parts into one harmonious whole.

He should have strong social feeling, so that all the relations of social life may be appreciated by him; so that his patients will become friends, and feel a lively interest in his success.

He should have continuity of purpose. All the great minds of the world have been marked by a spirit of continuance, a pushing ahead, and an unflagging zeal which could brook nothing less than ultimate success.

With an ordinary physical and mental man, yet with a great tenacity of purpose, a stick-to-it-iveness that never lets go, a fair degree of success may be attained. Yet, with all the faculties developed and a lack of application, failure must needs be the only result. Young man, cultivate a disposition to push to a finish any good work well begun!

Over and above all is his moral nature—that spirit of justice, that conception of right, that spiritual attitude toward God which enables him to see His hand in every created thing; who realizes that he is responsible to God for his every action, and that all his developments of physical and mental strength, and in all his perfection and 'beauty of manhood, he is nothing if he fails in his spiritual relation, in his moral training. For the physical must all pass away, and much of his mental training will go for naught. Then, with proper spiritual and moral training every lick he strikes will be for God and eternity.

He will feel that with the great Master-workman he is carrying out the Redemptive plan; that his life, while devoted to his profession, is, at the same time, making the world better; and as he perfects his own integrity of purpose, while he ascends to the highest point of development physically, mentally and morally, he will, at the same time, be elevating his profession, making it to stand, unblushingly, side by side with the grandest and best, and thus attain the fullest measure of manhood, assuming his right relationship toward God, his profession and his fellowmen.

